# Short-term Preservation of Porcine Oocytes in Ambient Temperature: Novel Approaches

**DOI:** 10.1371/journal.pone.0014242

**Published:** 2010-12-07

**Authors:** Cai-Rong Yang, De-Qiang Miao, Qing-Hua Zhang, Lei Guo, Jing-Shan Tong, Yanchang Wei, Xin Huang, Yi Hou, Heide Schatten, ZhongHua Liu, Qing-Yuan Sun

**Affiliations:** 1 State Key Laboratory of Reproductive Biology, Institute of Zoology, Chinese Academy of Sciences, Beijing, People's Republic of China; 2 College of Life Science, Northeast Agricultural University of China, Harbin, People's Republic of China; 3 Department of Veterinary Pathobiology, University of Missouri, Columbia, Missouri, United States of America; Baylor College of Medicine, United States of America

## Abstract

The objective of this study was to evaluate the feasibility of preserving porcine oocytes without freezing. To optimize preservation conditions, porcine cumulus-oocyte complexes (COCs) were preserved in TCM-199, porcine follicular fluid (pFF) and FCS at different temperatures (4°C, 20°C, 25°C, 27.5°C, 30°C and 38.5°C) for 1 day, 2 days or 3 days. After preservation, oocyte morphology, germinal vesicle (GV) rate, actin cytoskeleton organization, cortical granule distribution, mitochondrial translocation and intracellular glutathione level were evaluated. Oocyte maturation was indicated by first polar body emission and spindle morphology after *in vitro* culture. Strikingly, when COCs were stored at 27.5°C for 3 days in pFF or FCS, more than 60% oocytes were still arrested at the GV stage and more than 50% oocytes matured into MII stages after culture. Almost 80% oocytes showed normal actin organization and cortical granule relocation to the cortex, and approximately 50% oocytes showed diffused mitochondria distribution patterns and normal spindle configurations. While stored in TCM-199, all these criteria decreased significantly. Glutathione (GSH) level in the pFF or FCS group was higher than in the TCM-199 group, but lower than in the non-preserved control group. The preserved oocytes could be fertilized and developed to blastocysts (about 10%) with normal cell number, which is clear evidence for their retaining the developmental potentiality after 3d preservation. Thus, we have developed a simple method for preserving immature pig oocytes at an ambient temperature for several days without evident damage of cytoplasm and keeping oocyte developmental competence.

## Introduction

Oocyte preservation and transport of oocytes are important aspects of research and experimentation which oftentimes needs to be performed at a convenient time or place that is different from the site of oocyte collection. As an excellent animal model, preservation of porcine oocytes also benefits studies on human oocytes because of the many similarities between porcine and human oocytes regarding physiology and immunology [1]. Maintaining meiotic arrest during preservation is critically important to improve the quality of *in vitro* maturated oocytes.

At the present time, low temperature freezing and vitrification are the only practical methods for long-term preservation of oocytes and embryos. Conventional cryopreservation methods (slow-freezing) proved that porcine oocytes were highly sensitive to temperature below 15°C [2], and the formation of ice crystals from the lipid content of the cytoplasm was recognized as the main reason for the high sensitivity to low temperature [3]. Low temperature may also negatively affect subsequent nuclear and cytoplasmic reorganization of the GV stage oocytes [4] and even damage actin [5], mitochondria [6] and microtubules including those comprising the meiotic spindle [7]. In addition, cryoprotectant (CPA) toxicity and osmotic injury to the oocytes often occur during the thawing/warming phase [1]. These problems hampered the application of oocyte cryopreservation. Therefore, it is essential to have a new method available for oocyte preservation based on physiological conditions without adding drugs or thawing/warming while not damaging developmental competence of porcine oocytes.

Compared to spermatozoa, oocytes are more fragile and difficult to store without freezing [8]. However, there are reports on short-term preservation of oocytes without freezing. An early report showed that porcine oocytes still displayed maturation capability and developmental capacity after 24 h preservation at 20°C in TCM-199 [9]. A previous study also demonstrated that at least a few percent of mouse oocytes stored at room temperature retained the potential for full-term development, but irreversible injuries not only damage the cytoplasm but also the spindle apparatus [8]. Likewise, it was also reported that mouse oocytes could be preserved at room temperature and parthenogenetic developmental competence was not affected by exposure of oocytes to room temperature for 1, 2 or 4 h in Dulbecco's Phosphate Buffered Saline (DPBS) [10].

Although various drugs were able to maintain meiotic arrest efficiently, they are toxic to oocytes. Physiological methods have also been used to maintain oocyte meiotic arrest, but only for a limited time [11]. Hence, an oocyte preservation method that maintains an extended meiotic arrest at a lower temperature without toxic or damaging effects has not yet been developed.

Porcine follicular fluid (pFF) is an important ingredient of ovary and oocytes are kept at the GV stages in vivo in the follicular fluid environment for a long time before gondotropin surge. A previous study reported that pFF contains a factor(s) which could inhibit porcine oocyte maturation [12]. Fetal calf serum (FCS) can be used to transport mitotic cells at an ambient temperature without evidently damaging the survival of cells. In our experience, somatic cells can be stored in the FCS and transported at an ambient temperature for 4–5 days and then used for cell culture. The cells grow well after such a long time transportation at an ambient temperature. Therefore, we hypothesized that pFF and FCS could be used to preserve oocyte for a couple of days at an ambient temperature.

The objective of this study was to determine how long could the pig oocytes be preserved in the pFF and FCS without freezing at different temperatures. To assess the quality of preserved oocytes, COC morphology, germinal vesicle (GV) arrest, actin organization, glutathione (GSH) content, redistribution of CGs and mitochondria after preservation, maturation capability and spindle configuration after culture, and early developmental competence after in vitro fertilization were evaluated. Our study is the first to preserve immature porcine oocytes in two kinds of fluids (pFF and FCS) for 3 d.

## Results

### Effects of oocyte preservation temperature on inhibition of meiotic resumption in different media

When porcine oocytes were preserved in pFF and FCS for 3 d, 65% oocytes in pFF and 64% oocytes in FCS were arrested at the GV stage at 27.5°C. However, only 4% was arrested at the GV stage in TCM-199. Although meiotic arrest was maintained successfully for 3 d in either pFF or FCS at 27.5°C, pFF had a wider effective temperature interval (from 20°C to 30°C) than FCS (from 27.5°C to 30°C). There were almost no oocytes arrested at the GV stage at 4°C and 38.5°C (Table 1). The effects of different media, time, and the interaction between them on GV maintenance were all significantly different (P<0.05).

**Table 1:** Effects of temperatures and media on GVBD inhibition of porcine oocytes after preservation for 3 d*.

Temperature(°C)	Culture Medium					
	TCM-199		pFF		FCS	
	COC^1^	%^2^ GV	COC^1^	%^2^ GV	COC^1^	%^2^ GV
4	69	0±0^a^ ^A^	62	0±0^a^ ^A^	73	1.59±1.59^a^ ^A^
20	73	0±0^a^ ^A^	60	17.26±8.55^a^ ^B^	66	1.52±1.52^a^ ^A^
25	64	3.62±2.06^a^ ^A^	72	23.89±3.89^b^ ^B^	70	1.33±1.33^a^ ^A^
27.5	75	3.80±2.22^a^ ^A^	86	65.32±5.82^b^ ^D^	86	63.90±4.70^b^ ^C^
30	105	9.00±3.49^a^ ^A^	89	43.98±1.70^b^ ^C^	71	39.41±0.98^b^ ^B^
38.5	62	0±0^a^ ^A^	62	0±0^a^ ^A^	69	3.90±2.09^a^ ^A^

Data are presented as means ± SEM from three replicated experiments.

* Table1 was analyzed with double factor variance analysis, because the effects of different media, time, and the interaction are all significant. So we did not consider the main effects.

1 Means oocytes treated in every group.

2 Percentages are based on the total number of oocytes examined in every group.

A, B, C, D Values with different superscripts are significantly different in each column (P<0.05).

a, b Values with different superscripts are significantly different in each line (P<0.05).

### Effects of preservation time on inhibition of meiotic resumption

When porcine oocytes were preserved for different times (1 d, 2 d or 3 d) at 27.5°C, 74% oocytes in pFF and 71% in FCS were arrested at the GV stage for 1 d, while only 9% oocytes were arrested at the GV stage in TCM-199. The rate of oocytes at the GV stage dropped to 63% and 67% in pFF and FCS, respectively, after preservation for 2 d. There were no further significant changes in GV rates after preservation for 3 d (Table 2).

**Table 2:** The GVBD inhibition after preservation for different times in different media at 27.5°C.

Days	Culture Medium					
	TCM-199		pFF		FCS	
	COC^1^	%^2^GV	COC^1^	%^2^GV	COC^1^	%^2^GV
1	72	9.37±2.20^a^ ^A^	73	74.18±2.28^b^ ^A^	78	71.48±2.02^b^ ^A^
2	73	5.58±3.88^a^ ^A^	73	62.88±1.49^b^ ^B^	76	67.14±2.35^b^ ^AB^
3	77	3.57±2.06^a^ ^A^	83	60.38±1.99^b^ ^B^	66	62.20±1.71^b^ ^B^

Data are presented as means ± SEM from three replicated experiments.

1 Means oocytes treated in every group.

2 Percentages are based on the total number of oocytes examined in every group.

A, B Values with different superscripts are significantly different in each column (P<0.05).

a, b Values with different superscripts are significantly different in each line (P<0.05).

### Maturation of porcine oocytes after preservation in different media

There was no difference between the maturation rate of the oocytes preserved in pFF and FCS (57% and 52%). However, in both groups the maturation rate was lower than in the control (69%). Only 7% of oocytes preserved in TCM-199 for 3 d matured to the MII stages (Table 3).

**Table 3:** Maturation of oocytes after preservation at 27.5°C in different media.

	Oocytes examined	% matured oocytes
Control	94	68.70±1.42^a^
TCM-199	67	7.41±1.31^b^
pFF	73	56.68±2.53^c^
FCS	69	52.28±1.46^c^

Data are presented as means ± SEM from three replicated experiments.

a, b, c Values with different superscripts are significantly different in each column (P<0.05).

### Effects of different preservation media on GSH level

When porcine oocytes were preserved in pFF and FCS at 27.5°C for 3 d, the content of intracellular GSH was 1.35 and 1.59 pmol/oocytes, respectively, which was significantly higher than those preserved in TCM-199 with a low level of 0.07 pmol/oocytes, but still lower than in fresh oocytes probably due to the degeneration of parts of the oocytes preserved (Table 4).

**Table 4:** Effects of different preservation media on GSH level after preservation at 27.5°C for 3 d.

	Oocytes examined	GSH content (pmol/oocyte)
Control	90	2.94±0.06^a^
TCM-199	88	0.07±0.02^b^
pFF	90	1.35±0.23^c^
FCS	89	1.59±0.04^c^

Data are presented as means ± SEM from three replicated experiments.

a, b, c Values with different superscripts are significantly different in each column (P<0.05).

### Morphology of porcine oocytes after preservation in different media

**Figure 1:**
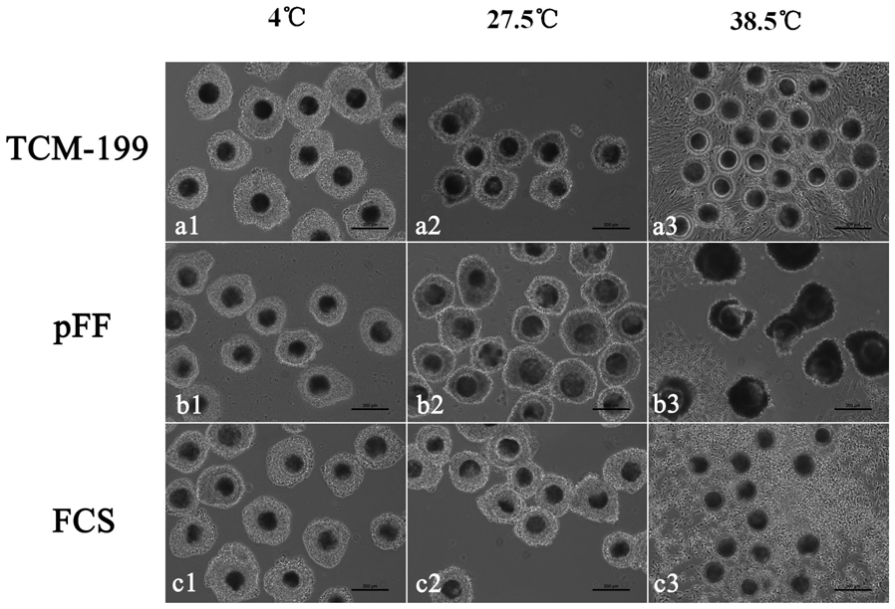
Morphologic changes of porcine cumulus-oocyte complexes (COCs) after preservation for 3 d. a1, a2, b1, b2, c1, c2, When preserved in TCM-199, pFF and FCS at 4°C or 27.5°C, cumulus cells attached tightly to oocytes and displayed no expansion; a3, c3, After preservation in TCM-199 and FCS at 38.5°C, almost all of the cumulus have shed from the oocytes; b3, After pFF preservation at 38.5°C, the whole COCs degenerated and showed black appearance; b2, c2, After preservation in pFF and FCS at 27.5°C, oocytes displayed dark ooplasm with compact cumulus cells. Scale bar  = 200 µm.

As shown in Fig. 1, cumulus cells attached tightly to oocytes and remained unexpanded when preserved at 4°C and 27.5°C for 3 d, but cumulus cells detached from oocytes or expanded if preserved in TCM-199 and FCS at 38.5°C for 3 d. Darkening of cytoplasm was observed in oocytes preserved in TCM-199 at 4°C, and COCs degenerated in pFF at 38.5°C. There was no expansion or proliferation of the cumulus cells preserved in pFF and FCS at 27.5°C.

### Effects of different preservation media on actin organizations

**Figure 2:**
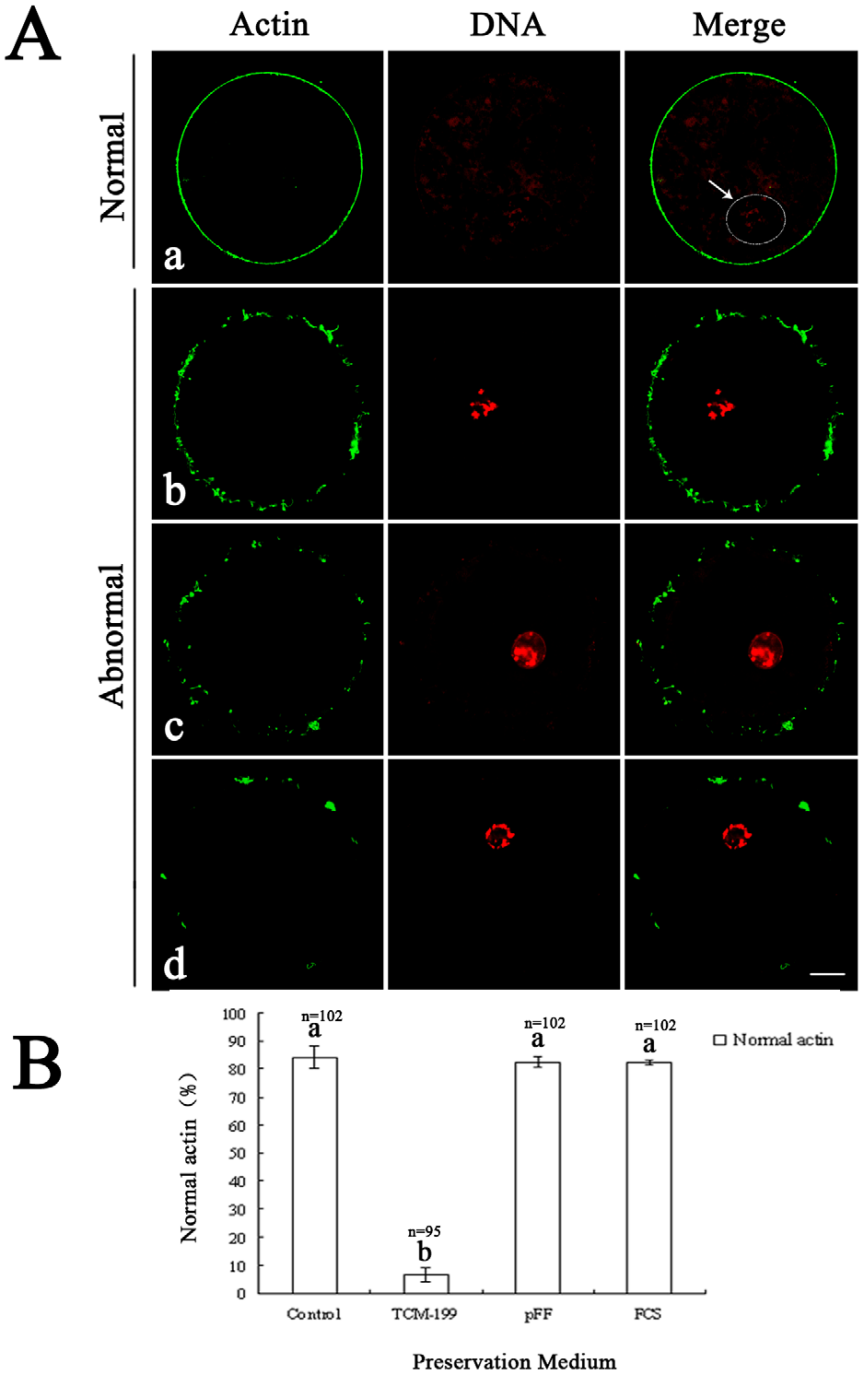
Effect of different media on actin configuration in porcine oocytes after preservation. A. a, represents the normal actin pattern in porcine oocytes (white arrow pointing at the white circle denotes the germinal vesicle). b, c, and d represent abnormal actin patterns. In b, actin fragments formed an incomplete ring with large punctiform aggregation. In c, small punctiform actin aggregated as an incomplete ring. In d, only some actin aggregated near oocyte membrane. Green, microfilaments; Red, chromatin. Scale bar  = 10 µm. B. Ratios of oocytes with normal actin configuration in control group and different preservation groups. Porcine oocytes were preserved in TCM-199, pFF and FCS at 27.5°C for 3 d, then the actin configuration was stained with FITC-phalloidine. Normal and abnormal actin configuration were detected. The graph shows the mean ± SEM of three independent experiments. The number “n” over the bars means the total treated oocyte in every group. The superscripts ^a, b^ over the bars represent values of normal actin configuration that differ significantly (P<0.05).

Two types of actin organizations were observed with laser confocal microscopy. Normal actin organization was prominently detected at the cell cortex of oocytes, while abnormal expression of actin showed discontinuous or incomplete distribution (Fig. 2A). Approximately 82% of oocytes showed normal actin distribution after preservation in pFF or FCS for 3 d, and only 7% of oocytes displayed intact distribution of actin in TCM-199. There was no significant difference between control oocytes and those preserved in pFF and FCS for 3 d (Fig. 2B).

### Effects of different preservation media on distribution of cortical granules (CGs)

**Figure 3:**
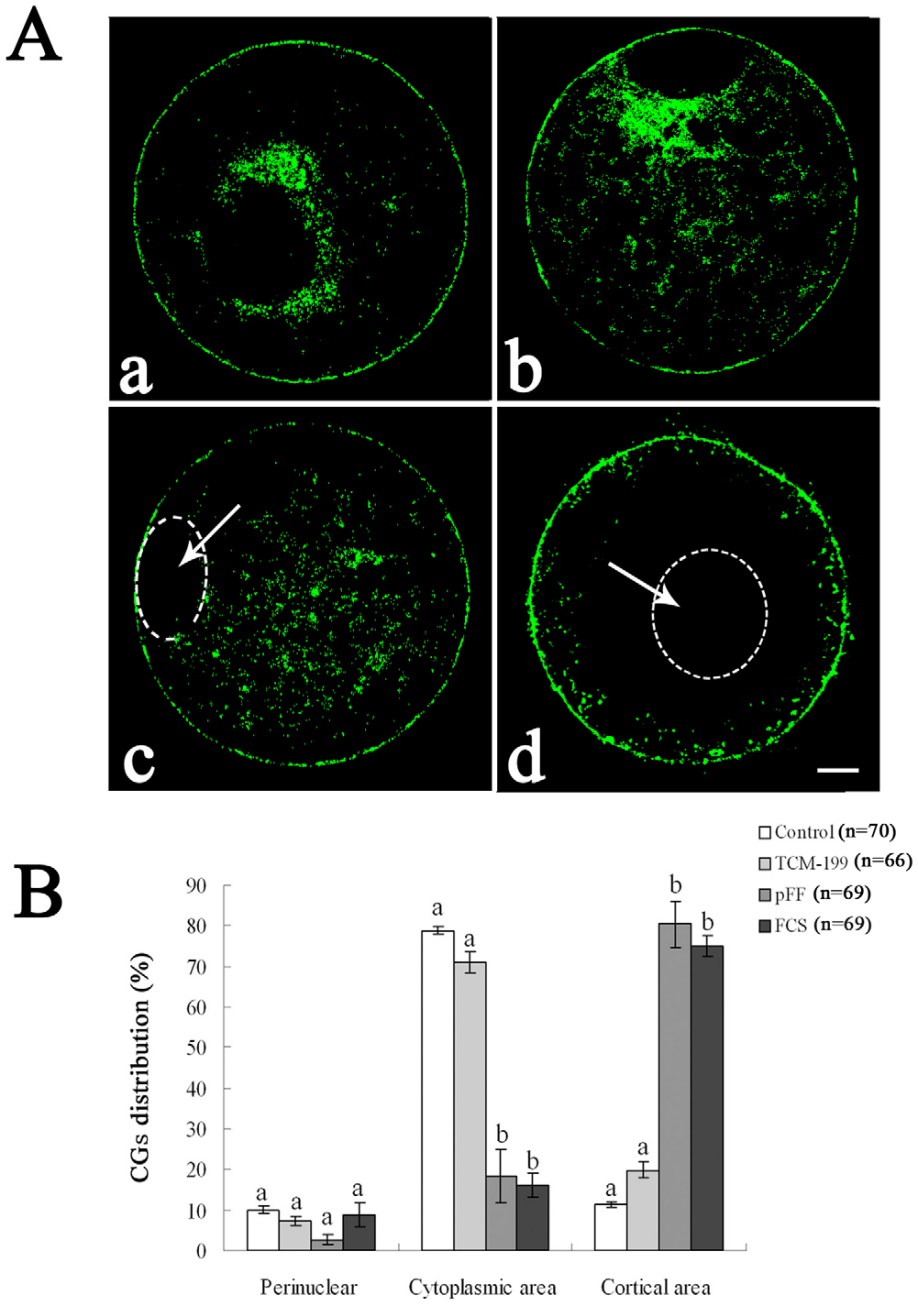
Effect of different media on CG distribution in porcine oocytes after preservation. A. a, b, CGs perinuclear area distribution within the porcine oocytes; a, most of the CGs aggregate in the perinuclear area, with a few migrating to the central inner cytoplasm or cortical area; b is intermediate pattern between a and c, with most of the CGs still remaining in perinuclear area and some migrating to the central cytoplasm; c, CGs distributed uniformly in the inner cytoplasmic area, and a few migrated to the cortical area to form a discontinuous ring; d, CGs cortical area distribution; almost all of the CGs have migrated into the cortical area, and a few still remained in the central cytoplasm. Green, cortical granules. Scale bar = 10 µm. (White arrows pointing at the white circle denotes the germinal vesicle). B. Ratios of different CG distribution patterns within the porcine oocytes after preservation in different media. Porcine oocytes were preserved in TCM-199, pFF and FCS at 27.5°C for 3 d, and then CG distribution was stained with FITC-labeled peanut agglutinin. Three categories of CG distribution were detected: perinuclear area; inner cyoplasmic area; cortical area. The graph shows the mean ± SEM of three independent experiments. The number “n” in the bracket means the total treated oocyte in every group. The superscripts ^a, b^ over the bars represent values that differ significantly in every categories of CG distribution (P<0.05).

When porcine oocytes were preserved in TCM-199, pFF or FCS at 27.5°C for 3 d, CGs were redistributed to different degrees. CG distribution was classified into three categories: a) CGs were mainly distributed around the germinal vesicles with a portion of CGs having migrated into the centre of the cytoplasm (Fig. 3A a,b); b) CGs were distributed uniformly throughout the oocyte cytoplasm including the cortex (Fig. 3A c); c) CGs were distributed at the cortex with few or no sporadic CGs still located in the inner cytoplasm (Fig. 3A d).

Seventy-nine percent of fresh oocytes showed CG distribution in the central cytoplasm, similar to oocytes preserved in TCM-199 (71%); however, only 18% of oocytes preserved in pFF and 16% of oocytes preserved in FCS showed CG distribution at the central cytoplasm. Most of the oocytes (80%, 75%) displayed cortical area distribution patterns in the pFF and FCS groups (Fig. 3B).

### Effects of different preservation media on mitochondria distribution

**Figure 4:**
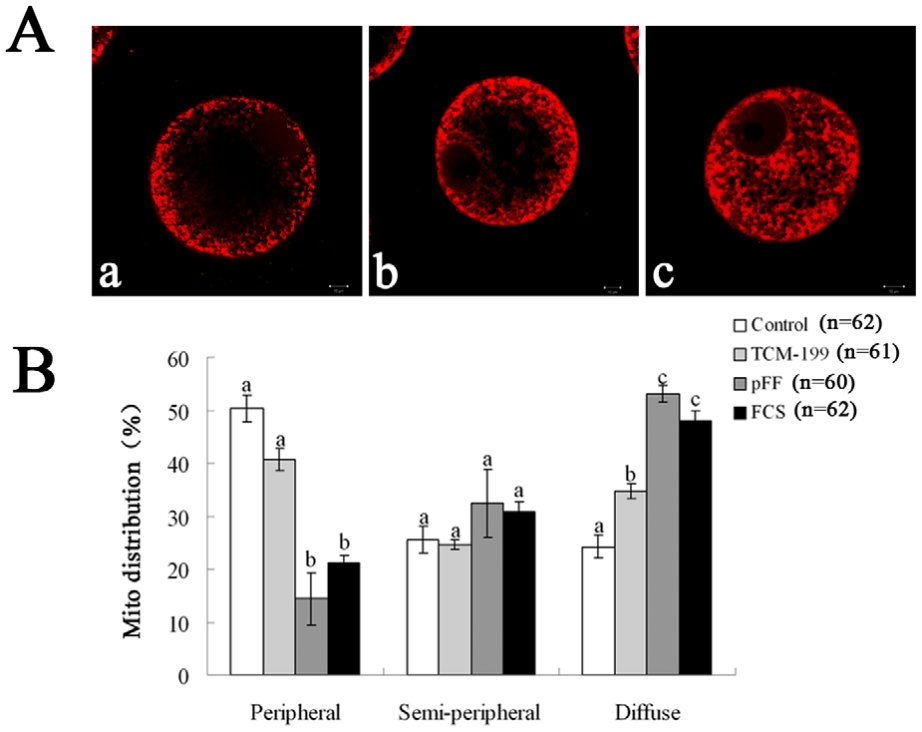
Effects of different media on mitochondria distribution in porcine oocytes after preservation. A. a, peripheral mitochondrial distribution, with no distribution at the center of the oocytes; b, semi-peripheral mitochondrial distribution; c, mitochondrial relocation and uniform distribution in the inner region of oocyte cytoplasm. Red, Mito Tracker Red staining. Scale bar  = 10 µm. B. Ratios of different mitochondria distribution patterns within the porcine oocytes after preservation with different media. Porcine oocytes were preserved in TCM-199, pFF and FCS at 27.5°C for 3 d, and then mitochondria distribution was stained with MitoTracker Red CMXRos. Three categories of mitochondria distribution were detected: peripheral; semi-peripheral; diffuse. The graph shows the mean ± SEM of three independent experiments. The number “n” in the bracket means the total treated oocyte in every group. The superscripts ^a, b, c^ over the bars represent values that differ significantly in every categories of mitochondrial distribution (P<0.05).

Three categories of mitochondrial distribution patterns were observed: a) oocytes showed strong staining of homogeneously distributed peripheral mitochondria (Fig. 4A a); b) some mitochondria were translocated into half of the cytoplasimc region in many oocytes, but still some of them were distributed in the peripheral area (Fig. 4A b); c) mitochondria were distributed homogeneously in the central cytoplasm (Fig. 4A c).

Half (50%) of the fresh oocytes showed peripheral distribution of mitochondria, while only 25% of oocytes showed semi-peripheral distribution or diffuse distribution. When oocytes were preserved in TCM-199, pFF or FCS, there was no difference in semi-peripheral mitochondria distribution patterns. However, approximately 50% of oocytes displayed diffuse distribution in the pFF (53%) and FCS (48%) groups, while only 35% of oocytes in the TCM-199 group and 24% of oocytes in the control group showed such a pattern (Fig. 4B).

### Effects of different preservation media on spindle configuration in porcine oocytes after in vitro maturation (IVM)

**Figure 5:**
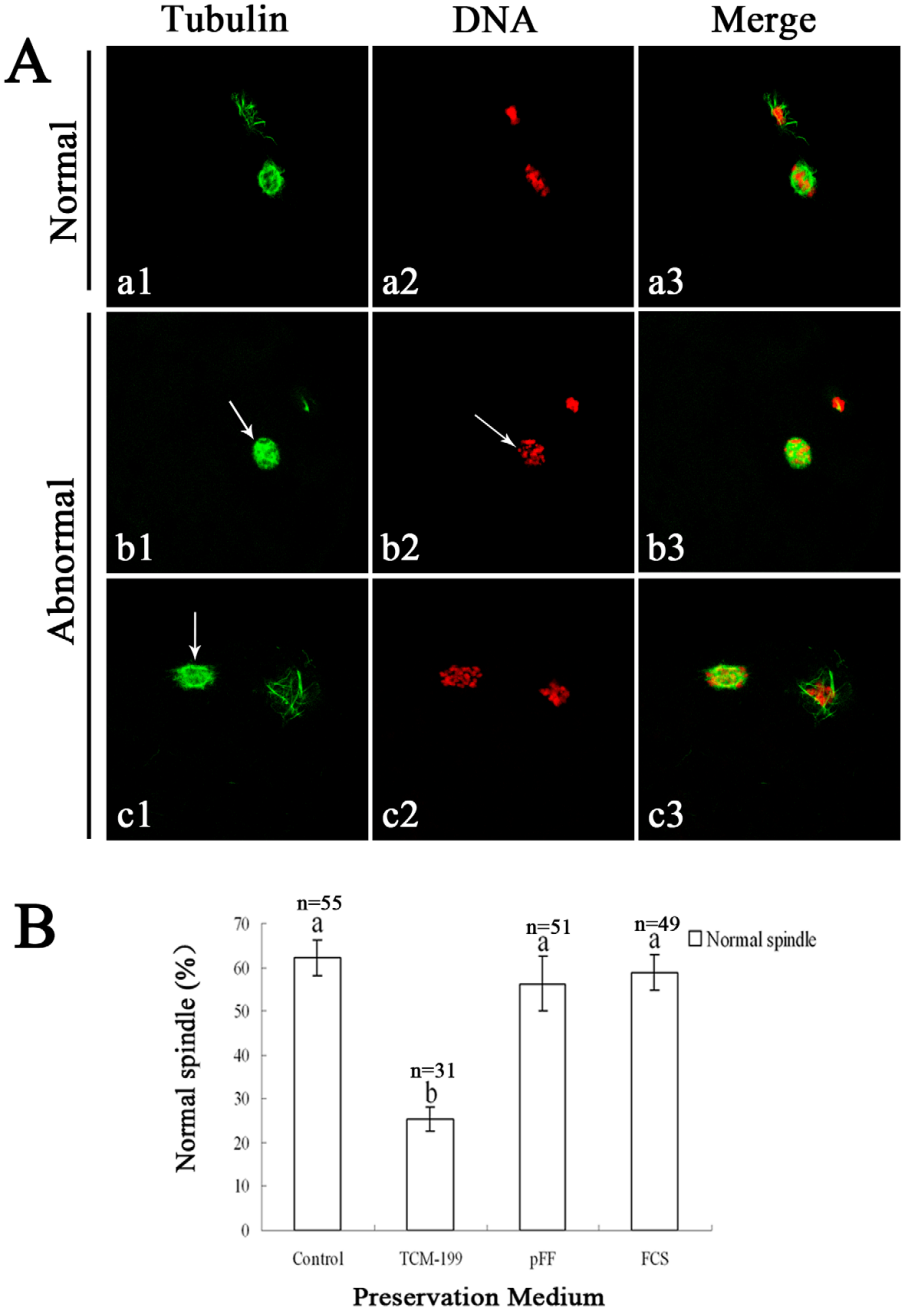
Effects of different preservation media on spindle configuration in porcine oocytes after IVM. A. a, represents a normal spindle in porcine oocyte. b and c represent abnormal spindle. In b1, c1 arrows indicate abnormal spindle organization distribution. In b2, arrows indicate fragmented and displaced chromosomes. Green, tubulin; Red, chromatin. Scale bar  = 10 µm. B. Normal ratios of spindle configuration in porcine oocytes after preservation in different media following IVM. Porcine oocytes were preserved in TCM-199, pFF and FCS at 27.5°C for 3 d, then spindle was stained with FITC-α-tubulin after culture for 44 h *in vitro*. Normal and abnormal spindle configuration were detected. The graph shows the mean ± SEM of three independent experiments. The number “n” over the bars means the total treated oocyte in every group. The superscripts ^a, b^ over the bars represent values of normal tubulin configuration that differ significantly between groups (P<0.05).

The normal spindle was barrel-shaped, with regular chromosomal alignment, while abnormal spindle configurations showed disorganized microtubules along with scattered or fragmented chromosomes (Fig. 5A). Almost 60% oocytes displayed normal spindle configurations in the pFF and FCS groups, while only 25% oocytes displayed normal spindle configurations in the TCM-199 group. There was no significant difference between the control group and the pFF group or FCS group (Fig. 5B).

### Effects of different preservation media on early embryo development

After preserving in different media, porcine oocytes were cultured *in vitro*. Following in vitro fertilization (IVF), there was no difference between the cleavage rate of the oocytes preserved in pFF and FCS (56% and 60%, respectively) (P>0.05). However, significant differences were found in preserved groups and control group (72%) (P<0.05). Only 5% of oocytes preserved in TCM-199 for 3 d developed to 2-cell stage after IVM-IVF. However, there was no oocyte developed to the blastocyst stage in TCM-199 group. The incidence of embryos developing to the blastocyst stage in pFF (10%) or FCS (12%) group was lower than that of the control group (28%) (P<0.05). Although the mean cell numbers per blastocyst were 35 and 37, respectively in pFF and FCS groups, lower than the control, there was no significant difference between them (P>0.05) (Table 5).

**Table 5:** Effect of preserving medium on early development of IVM-IVF porcine oocytes after preservation at 27.5°C for 3 d.

Treatment	Oocytes examined	%^1^ of oocytes cleaved (n)	% 1 of blastocysts formation(n)	No. cells/blastocyte
Control	227	71.95±2.04^a^ (164)	27.97±1.38^a^ (64)	40.67±1.76^a^
TCM-199	28	5.29±2.90^b^ (3)	0±0^b^ (0)	0±0^b^
pFF	183	56.49±3.21^c^ (103)	10.20±0.89^c^ (19)	35.33±1.45^a^
FCS	209	59.68±4.41^c^ (125)	11.79±1.28 c (25)	36.67±1.20^a^

Data are presented as means ± SEM from three replicated experiments.

The values “n” in bracket represent the number of the oocytes cleaved at 48 hr and forming blastocysts at 7 d after IVF.

1 Percentages are based on the total number of oocytes examined.

a, b, c Values with different superscripts are significantly different in each column (P<0.05).

## Discussion

It is generally accepted that porcine oocyte preservation is highly significant for agriculture and biomedicine. It also has implications for human assisted reproduction technologies. However, many problems still exist concerning conventional porcine oocyte preservation methods, such as low temperature sensitivity, cryoinjuries, toxicity of preservation solution and low success rates. Therefore, it is important to determine new methods for short-term oocyte preservation and long distance transportation, which not only avoids toxicity and cryoinjuries, but also allows synchronous maturation. Here, we report that 27.5°C is the optimal temperature for preservation of oocytes in pFF or FCS. After preservation for 3 d, oocytes are still viable, remain at the GV stage, and have undergone beneficial cytoplasmic changes.

Our study has addressed three questions. The first question involves in the temperature sensitivity and preservation medium. The second question relates to oocyte quality after preservation. The third aspect deals with synchronization of nuclear and cytoplasmic maturation.

Temperature and medium are very important for oocyte preservation. It has been shown that offspring could be produced from mouse oocytes stored at room temperature in TYH, CZB-H and KSOM media with 0 to 0.5% Bovine serum albumin (BSA), not from oocytes stored at 5 or 37°C [8]. Compared with other species, porcine oocytes and embryos are more sensitive to low temperature [13]. Previous study demonstrated that the survival rate of porcine oocytes stored at 20°C was higher than at 37°C in TCM-199 with serum after 48 h [9]. Our results showed that 27.5°C was the optimal temperature for storing porcine oocytes in pFF or FCS. Porcine immature oocytes in the TCM-199 group showed the lowest rates of GVBD inhibition and further development to the MII stage after culture, while preservation of oocytes in pFF or FCS for 3 d at 27.5°C achieved significant GVBD inhibition and more than half of the oocytes completed nuclear maturation after culture. Follicular fluid and follicular shell fragments in the culture medium not only promote cytoplasmic maturation, but also contain a factor(s) which could inhibit porcine oocyte maturation [12]. Porcine oocytes were previously preserved by using pFF. After 4 h storage at room temperature, oocytes showed the lowest rates of cleavage and development to the morula/blastocyst stages after fertilization, because follicular fluid was sticky and turbid [10]. In our study pFF was centrifuged, and only the supernatant was used for preservation, so these problems were avoided, and oocytes can survive for 3 d. Our results indicate that FCS is also an appropriate medium for oocyte preservation at 27.5 °C.

We next determined the oocyte quality after preservation on subcellular levels. Actin organization, CG migration, GSH content and mitochondria redistribution and spindle appearance were regarded as indicators of oocytes quality. It is well known that dynamic regulation of the actin cytoskeleton is important for maintenance of cellular structures and specific cellular functions [14], [15]. When F-actin polymerization is inhibited, completion of oocyte meiotic maturation and embryo development are prevented [16]. Traditional cryopreservation methods (vitrification) cause a decrease in the percentage of oocytes with normal distribution of F-actin in both GV and metaphase II (MII) stage oocytes [5]. Our results proved that the percentage of oocytes with normal distribution of F-actin was similar in GV stages among fresh, pFF and FCS groups, indicating that pFF and FCS do not disturb actin organization.

For many species, CG migration and mitochondrial distribution are regarded as clear indications of oocyte cytoplasmic maturation [17]. During oocyte growth and maturation, CGs migrate to the cortex and form a continuous layer underneath the oolemma [18], [19]. Any dysfunction or dislocation of cortical granules would decrease oocyte competence and has detrimental effects on embryo quality [20]. Our results indicate that most of the CGs migrated to the cortical area when oocytes were preserved in pFF or FCS for 3d. The migration of CGs toward the cortical region in oocytes preserved in the pFF or FCS group is a beneficial change for cytoplasmic maturation.

The activity and organization of mitochondria are necessary features among the diverse events involved in oocyte cytoplasmic maturation [21], and the homogeneous distribution of mitochondria in the cytoplasm is positively correlated with ATP content [22]. Dysfunction of mitochondria often appeared in vitrified oocytes. It was reported that the rate of oocytes with abnormal mitochondria distribution was increased after vitrification [6]. Indeed, in the pig, mitochondria were restricted to the oocyte periphery at GV stages, but migrated to the inner region of the cell during maturation both *in vitro* [23], [24] and *in vivo* [25]. The relocation of mitochondria occurs in oocytes with high developmental competence [26], [27]. In contrast, little or no relocation was detected in low competence oocytes in which mitochondria persisted in a peripheral distribution pattern [28]. All these features suggest a linkage between low developmental ability and perturbed mitochondrial distribution. In our study, about half of the fresh oocytes showed peripheral distribution of mitochondria, while only one quarter showed semi-peripheral distribution or central cytoplasm distribution. On the contrary, approximately half of the oocytes displayed central cytoplasm distribution patterns in oocytes preserved in pFF or FCS for 3 d, indicating progressive cytoplasmic maturation during the preservation period.

Cytoplasmic GSH content was also examined. GSH has been shown to be one of the markers for ooplasm quality. It regulates sperm nuclear decondensation and male pronuclear formation [29] and it protects cells against oxidative damage [30]. Low intracellular GSH may be responsible, in part, for the low developmental competence of porcine oocytes [31], [32]. Our study shows that GSH content is decreased after preservation in pFF or FCS when compared to fresh oocytes, and that the TCM-199 group shows lowest GSH levels. The decrease in GSH content in preserved oocytes may be due to the degeneration of some preserved oocytes, but more than half of the oocytes maintained viability and developed into MII stages after 3 d preservation.

Oocytes need to undergo cytoplasmic maturation as well as nuclear maturation to become able to support successful development [33]. Synchronized cytoplasmic maturation and nuclear maturation are considered important for normal embryo development after fertilization [34]. A premature meiotic resumption without adequate cytoplasmic maturation was induced when oocytes were transferred into culture medium from follicles, which was one of the main reasons for poor quality. The peripheral migration of CGs and interior translocation of mitochondria during the preservation period may contribute to synchronization of cytoplasmic and nuclear maturation. Previous study found that the percentage of oocytes with normal spindle organization was decreased when vitrified GV oocytes were cultured to assess maturation [5]. Our results show that the percentage of normal spindles was not affected by preserving oocytes in pFF or FCS compared with the control group. Further studies have already clarified that oocytes preserved for 3 d in pFF or FCS have abilities to develop to term after IVM and IVF.

Taken together, our studies show that porcine oocytes could be preserved in pFF or FCS at 27.5°C for 3 d without evident cytoplasmic damage. More than half of the preserved oocytes retain the potential to mature *in vitro*, with normal spindle formation, and about 10% oocytes developed to blastocysts with normal cell numbers after in vitro fertilization. Thus, we have developed a simple method for short-term (up to 3 d) preservation of porcine oocytes *in vitro*. Further research is necessary to clarify whether these preserved oocytes can produce piglets after IVF.

## Materials and Methods

### Ethics statement

Porcine handling was conducted in accordance with policies promulgated by the Ethics Committee of the Institute of Zoology, Chinese Academy of Sciences. Porcine ovaries used in this study were obtained from AnDing slaughterhouse, a local slaughterhouse of Beijing, P.R.China.

### Chemicals and antibodies

All chemicals used in this study were purchased from Sigma Chemical Company (St. Louis, MO), unless otherwise noted. The GSH and GSSG Assay Kit was purchased from Beyotime Institute of Biotechnology (Beyotime, CN). Fetal calf serum was purchased from Gibco (Gibco, Grand Island, NY).

### Preparation of pFF and FCS

pFF was prepared as follows: 1) The contents of follicles measuring 3–5 mm in diameter were aspirated with an 18-gaugle needle fixed to a 20-ml disposable syringe and pooled into a 50 ml conical tube (Falcon, Franklin Lakes, NJ). After sedimentation for about 15 min, the pellet was discarded; 2) The supernatant was centrifuged at 15,000 rpm for 15 min, then the supernatant was collected and frozen at −20°C. After thawing, the fluid was centrifuged followed by two cycles of freezing and thawing, each time discarding the pellet; 3) The supernatant was collected and filtrated for sterilization, and then stored at −20°C until use.

FCS was purchased from Gibco (Grand Island, NY), and was subpackaged in 2-ml tube, then stored at −20°C until use.

### Collection and preservation of porcine oocytes

Porcine ovaries were obtained from prepubertal gilts at a local slaughterhouse and transported to the laboratory within 1 hour. The ovaries were maintained in 0.9% NaCl solution containing penicillin G (75 mg/mL) and streptomycin sulphate (50 mg/mL) at 34°C ∼36°C for the entire time of the trip. Cumulus-oocyte complexes (COCs) were aspirated from antral follicles (3–6 mm in diameter) of ovaries with an 18-gauge needle fixed to a 20 ml disposable syringe.

After three rinses in washing medium (TCM-199 medium supplemented with 2.2% NaHCO_3_), COCs with uniform cytoplasm and compact cumulus mass were selected for preservation. The COCs were washed three times in 50 µl droplets of different fluids, then groups of 25 to 30 COCs were preserved in a 200 µl drop of TCM-199, pFF and FCS at different preservative temperatures (4°C,20°C,25°C,27.5°C,30°C,38.5°C) in air condition, covered with liquid paraffin oil in a biochemical incubator. The COCs were preserved for 1 d, 2 d or 3 d. Evaluation of morphological survival (homogenous cytoplasm, intact and compact cumulus mass) was carried out after preservation.

### In vitro maturation (IVM)

The IVM of COCs was performed in accordance with the methods of our previous study [17], [35], [36], [37]. The basic medium was TCM-199, supplemented with 75 µg/ml potassium penicillin G, 50 µg/ml streptomycin sulphate, 0.57 mM cysteine, 0.5 µg/ml FSH, 0.5 µg/ml LH, and 10 ng/ml EGF. Approximately 40 to 50 COCs were cultured in a 200 µl drop of maturation medium in 4-well dishes, which was covered by liquid paraffin oil for up to 44 h at 39°C in an atmosphere of 5% CO_2_ in air and saturated humidity.

### Evaluation of GSH

Intracellular content of GSH was measured as described by instruction supplied with the GSH and GSSG Assay Kit. Briefly, after preservation, cumulus cells were removed completely from oocytes by vortex and washed three times in phosphate buffered solution (PBS). Thirty oocytes composed one sample and three samples were assayed for each treatment. Oocytes were washed two times with distilled water, then 5 µl of 0.2 M sodium phosphate containing 10 mM Na_4_-EDTA and 5 µl of 1.25 M phosphoric acid (Fluka, Buchs, Switzerland) were added to the 1.5 mL centrifuge tube containing 30 oocytes. Samples were vortexed thoroughly, and then frozen at −70°C and thawed at 37°C in a water bath. This procedure was repeated at least three times for dissolving oocytes. Then the samples were placed in an ice bath for 5 minutes and centrifuged at 10,000 rpm for 10 minutes at 4°C. The tubes containing samples were stored at −70°C until assayed. All samples and solution preparations were carried out according to the directions of Total Glutathione Assay Kit (BiYunTian, The Institute of Biotech, S0052, Shanghai, China). Concentrations of GSH in the oocytes were determined by the 5, 5′ dithio-bis (2-nitrobenzoic acid)-glutathione disulfide (DTNB-GSSG) reductase-recycling assay. Briefly, GSH content was measured according to methods described by Funahashi et al. [38] with minor modifications. First, 150 µl of detection solution was added to 96-well plates, and then 10 µl of samples was pipetted into each well. After the dishes were equilibrated at 25°C for 5 minutes, a total of 50 µl 0.16 mg/mL NADPH was added to each well. The formation of 5-thio-2-nitrobenzonic acid, which has an absorption peak at 412 nm, was monitored continuously with a spectrophotometer for 25 minutes with reading recorded every 5 minutes. Standards (0.5, 1, 2, and 10 µM) of glutathione and a sample blank lacking glutathione were also assayed at the same time. The amount of glutathione in each sample was divided by the number of oocytes [39].

### Assessment of nuclear status of porcine oocytes by orcein staining

After porcine COC preservation, oocytes were denuded of cumulus cells by vortexing, and then denuded oocytes were mounted on glass slides. We used vaseline and lanolin to keep the coverslip in contact with the oocytes without extensive pressure. For fixation, the slides were immersed in 25% (v/v) acetic acid in ethanol for at least 48 hours at room temperature (25°C). Oocytes were stained with 1% (w/v) orcein in 45% (v/v) acetic acid and examined under a light microscopy at a magnification of ×100. Nuclear stages were clearly observed, oocytes containing filamentous or condensed chromatin enclosed in a nuclear envelope were classified as germinal vesicle (GV) stage. Oocyte maturation status after culture was also evaluated using the same protocol.

### Evaluation of actin by confocal laser scanning microscopy

For microfilament detection, the zona pellucida were first removed from oocytes in acid Tyrode's solution (pH 2.5), and then oocytes were fixed with 4% paraformaldehyde in PBS for at least 30 minutes at room temperature. They were permeabilized in PBS containing 1% Triton X-100 overnight at 37°C, followed by blocking in PBS containing 1% BSA (blocking solution) for 1 h at room temperature. After washing in PBS containing 0.1% Tween 20 and 0.01% Triton X-100 (washing solution) for 15 minutes, the oocytes were stained with FITC-phalloidin diluted 1∶100 with washing solution for 2 hours at room temperature in a dark box. Then after washes in washing solution, nuclear status was determined after staining with PI (propidium iodide, 10 µg/ml in PBS) for 10 minutes. Oocytes were washed extensively, mounted on glass slides, and observed under a laser-scanning confocal microscope (Zeiss LSM 510 META, Germany). At least 25 oocytes were examined for each time point, and each treatment was repeated at least three times.

The scorning system of actin was based on the method described in a previous study[40]. Two types of actin organizations were observed with laser confocal microscopy. Normal actin organization was prominently detected at the cell cortex of oocytes, while abnormal expression of actin showed discontinuous or incomplete distribution.

### Evaluation of cortical granules by confocal laser scanning microscopy

The method for staining CGs was based on the procedures reported in our previous study [41]. After removing the zona pellucida in acidic Tyrode's solution (pH 2.5), oocytes were fixed with 4% (w/v) paraformaldehyde in PBS for at least 30 minutes at room temperature, followed by washing in blocking solution (PBS containing 0.3% BSA and 100 mM glycine) three times for 5 min each. After 5 minutes of permeabilization in PBS containing 0.1% Triton X-100, oocytes were washed two additional times in blocking solution (5 minutes each). They were then labeled with 100 µg/ml FITC-labeled peanut agglutinin (Sigma) in PBS for 30 minutes in a dark box. Finally, the oocytes were washed three times in PBS containing 0.3% BSA and 0.01% Triton X-100. Nuclear status of the oocytes was determined after staining with 10 µg/ml PI in PBS for 10 minutes, followed by washing three times in PBS, and then mounted on glass slides. Oocytes were observed under a laser-scanning confocal microscope (Zeiss LSM 510 META, Germany). A total of 25 oocytes from each sample were scanned and recorded with confocal fluorescent microscopy. Each treatment was repeated at least three times.

The scorning system of cortical granule was based on a previous report [42]. Cortical granule migration was classified with the following categories: CGs were mainly distributed around the germinal vesicles with a small number of CGs having migrated into the centre of the cytoplasm; CGs were migrated throughout the oocyte cytoplasm uniformly including the cortex; CGs having migrated into the cortex with few or any sporadic CGs still distributed in the inner cytoplasm.

### Evaluation of mitochondrial distribution by confocal laser scanning microscopy

Oocytes were stained for mitochondria according to the methods of our previous study [17]. A stock solution of MitoTracker Red CMXRos (Molecular Probes, Eugene, OR) fluorescence probe at a concentration of 1 mM was prepared in dimethyl sulfoxide and stored at −20°C. Denuded oocytes at the GV stages were incubated in TCM-199 supplemented with 0.5 µmol/L MitoTracker Red CMXRos in a dark environment and humidified air with 5% CO_2_ for 30 minutes at 38.5°C, followed by three washes with TCM-199 for 20 minutes, and then observed with a laser-scanning confocal microscope (Zeiss LSM 710 META, Germany).

The scorning system of mitochondrial was based on a previous report [28]. Three categories of mitochondrial distribution patterns were observed: a) oocytes showed strong staining of homogeneously distributed peripheral mitochondria; b) some mitochondria were translocated into half of the cytoplasimc region in many oocytes, but still some of them were distributed in the peripheral area; c) mitochondria were distributed homogeneously in the central cytoplasm.

### Evaluation of spindle by confocal laser scanning microscopy

After maturation, only oocytes with a visible polar body were chosen for spindle detection. Oocytes were stained for spindle detection according to the methods of our previous studies [36], [43]. Cumulus-free oocytes were fixed with 4% paraformaldehyde in PBS for at least 30 minutes at room temperature. They were permeabilized in PBS containing 1% Triton X-100 overnight at 37°C, followed by blocking in PBS containing 1% BSA (blocking solution) for 1 hour at room temperature. After washing in PBS containing 0.1% Tween 20 and 0.01% Triton X-100 (washing solution) for 15 minutes, oocyte microtubules were labeled by incubation in FITC-conjugated monoclonal anti-α-tubulin antibody (Sigma) diluted 1∶100 with washing solution for 1 hour at room temperature in a dark box. After three washes in washing solution, nuclear status was determined after staining with PI (propidium iodide, 10 µg/ml in PBS) for 10 minutes oocytes were washed extensively, and mounted on glass slides. Oocytes were observed under a laser-scanning confocal microscope (Zeiss LSM 510 META, Germany). Each treatment was repeated at least three times.

### In vitro fertilization

Freshly ejaculated sperm-rich fraction was collected from fertile boars, following short incubation at 39°C, the semen was resuspended and washed 3 times in DPBS supplemented with 0.1% (w/v) BSA by centrifugation at 1500rpm for 4 min. The spermatozoa concentration was measured by a hemocytometer and the proportion of motile sperm was determined. The spermatozoa were diluted with modified Tris-buffered medium (mTBM) to optimal concentration. Cumulus-free matured oocytes were washed three times in mTBM and placed in prepared mTBM drops, covered with a mineral oil and incubated for 2 h at 38.5°C under 5% CO_2_ in air. Approximate 30 oocytes were inseminated in 50 µl drops at a final sperm concentration of 3×10^5^ cells/ml for 5 hr co-incubation.

At 5 h after insemination, the oocytes were incubated in 200 µl of PZM-3 in a 4-well culture plate for 7 d at 38.5°C in an atmosphere of 5% CO_2_ in air. Cleavage and blastocyst formation of the oocytes were examined at 2 and 7 d after the start of culture, respectively. The blastocysts were stained with 5 µg/ml bisbenzimide (Hoechst 33342) to determine the number of nuclei by using a fluorescent microscope.

### Statistical analysis

All data were obtained from at least three replicate trials for each experiment. All percentages from three repeated experiments were expressed as mean ± SEM. The percentage data were subjected to arc-sine transformation and the transformed data were tested to verify ANOVA assumptions before being analyzed with ANOVA. All statistics were performed with analysis of variance and Fisher's LSD analysis by SPSS version 10.0 (Installshield Software Corporation) except for table1. Table1 was analyzed with double factor variance analysis, because the effects of different media, temperatures and the interactions are all significant, so main effect was ignored. Different values were considered significant when the P value was less than 0.05.
